# Treatment efficacy analysis of traditional Chinese medicine for novel coronavirus pneumonia (COVID-19): an empirical study from Wuhan, Hubei Province, China

**DOI:** 10.1186/s13020-020-00317-x

**Published:** 2020-04-15

**Authors:** Erdan Luo, Daiyan Zhang, Hua Luo, Bowen Liu, Keming Zhao, Yonghua Zhao, Ying Bian, Yitao Wang

**Affiliations:** 1 State Key Laboratory of Quality Research in Chinese Medicine, Institute of Chinese Medical Sciences, University of Macau, Macao, China; 2 The First Affiliated Hospital of Liaoning University of Traditional Chinese Medicine, Shenyang, China; 3 Present Address: Physician in the China Medical Treatment Expert Group to support for Covid-19 Control in Jihe Hospital, Caidian District, Wuhan, Hubei province China

**Keywords:** Novel coronavirus pneumonia, Traditional Chinese Medicine, Treatment efficacy, Empirical study, Wuhan China

## Abstract

**Background:**

A novel coronavirus was identified in December, 2019 in Wuhan, China, and traditional Chinese medicine (TCM) played an active role in combating the novel coronavirus pneumonia (NCP) caused by this fast-spreading virus COVID-19. Thus, we aimed to explore TCM characteristics of clinical efficacy to NCP, as well as to optimize Qingfei Paidu decoction (QFPDD) and the recommended formulas to NCP by National Health Commission (NHC).

**Methods:**

Chinese medical sciences theory and clinical application of TCM were analyzed. A total of 54 NCP patients were observed in a hospital from Wuhan, whose clinical characteristics and utilization of Chinese Medicines (CMs) were described. Paired t test was used to measure the change of patients’ hemogram during hospitalization period, indicating the effect of CMs. Multiple linear regression analysis was applied to explore the factors affecting the length of hospital stay. Network pharmacology analysis was applied to figure out the performance of NHC-recommended formulas of five disease stages at levels of compounds, targets and pathways.

**Result:**

The average length of hospital stay was 8.96 days. Patients over 45 stayed 9.79 days in hospital in average, longer than 7.64 days of patients under 45. Comparing the hemograms between admission and discharge of hospital, the number of leukocytes, neutrophil, lymphocyte and platelet increased, while the numbers of erythrocytes, hemoglobin concentration and hematocrit decreased. According to the standard coefficients of regression, the factor affecting the length of stay for the most was CMs in category of invigorating spleen and removing dampness (ISRD), followed by administrating CMs, male, and cough. Thirty-two CMs were screened after deleting duplication from QFPDD and NHC-recommended formulas. Compound quercetin, luteolin, kaempferol, acacetin etc., were all involved in the treatment of various disease stages on the compound level both in generality and individuality.

**Conclusion:**

TCM has a systemic theoretical understanding on the pathological evolution and a positive clinical efficacy on NCP. The CMs of ISRD improved patients’ recovery, suggesting the importance of regulating intestinal function and keeping microenvironmental balance in TCM treatment of NCP. The active compounds from QFPDD and NHC-recommended formulas contribute to recovery of varied disease progresses during TCM treating NCP.

## Background

A novel coronavirus named COVID-19 was identified in December 2019 in Wuhan, China, which caused infectious pneumonia and spread rapidly. However, there has been no consensus on the nomenclature of novel coronavirus pneumonia (NCP) from the perspective of traditional Chines Medicine (TCM) so far. Academician Tong Xiaolin suggested that the disease should be named as cold-dampness pestilence (寒湿疫) [1], and academician Wang Qi called the disease pulmonary pestilence (肺瘟) in the Manual for Traditional Chinese Medicine diagnosis and treatment of NCP [2]. In general, there is an agreement on the opinion that NCP belongs to the category of epidemic disease (疫病) in TCM. As the special climate of Wuhan, where the local temperature in last winter was higher than that in previous winter, and the rainfall was more frequent than snowfall, the syndromes of NCP patients often presented the characteristics of dampness pathogen (湿邪) in TCM. Integrating the analysis resulted from Professor Liu Qingquan and Dr. Xiang Qiong [3, 4], we consider that NCP (COVID-19) should be defined as dampness toxin pestilence (湿毒疫). Dampness toxin (湿毒) runs through the comprehensive pathology of NCP. Even in Gansu, a region with dry climate, the researchers found that the characteristics of dampness pathogen from NCP patients were similar to those in Wuhan [5].

Chinese Medicine (CM) has accumulated abundant clinical experiences and effective formulas on the prevention and treatment of epidemic diseases. In Ming dynasty, Wu Youke, a famous Chinese medicine doctor, believed that the pathogen of epidemic disease was different from the six excesses (六淫), but was a kind of pestilent Qi (疠气) that had high contagious and powerful toxic features. Pestilent Qi is prone to encroaching specific organs and involving multiple organs failure, and commonly breaks out in populated large cities. In 2004, a clinical study including 524 patients with severe acute respiratory syndrome (SARS) showed that the duration of major symptoms in the group of patients treated by integrated Chinese and western medicines was significantly shorter than those in the group treated by western medicine alone [6]. The satisfied therapeutic effects of TCM in preventing and treating SARS suggested the superiority of TCM on severe infectious diseases.

In March 2020, the Diagnosis and Treatment Guideline of Novel Coronavirus Pneumonia (Edition 7) was released by the National Health Commission (NHC) of People’s Republic of China [7], in which Qingfei Paidu decoction (QFPDD) and other TCM formulas were recommended to treat NCP. Although it is necessary to consider the real pathological evolutions of patients based on local climatic features and individual physical characteristics of patients, the inconsistency of syndrome types (证型) is prone to producing cluttered Chinese medicine formulas. In the view of this, the TCM symptom types in this study complied with those in the guideline from treatment to recovery period.

Therefore, this study aimed to figure out efficacy of TCM in treating NCP, to explore the relationship of the TCM’s influence factors with patient’s individual characteristics, and to optimize QFPDD and NHC-recommended formulas corresponding to the treatment and recovery period of NCP.

By using both statistics analysis and network pharmacological technology, this study can not only partially reveal the therapeutic mechanisms of TCM through the corresponding relationships among formula, medicine, and syndrome, but also provide scientific evidence for screening and optimizing TCM formulas for the treatment of NCP.

## Methods

### Study population

Data of 54 patients with NCP, namely SARS-CoV-2 pneumonia (COVID-19) undergoing CMs treatment originated from the department of infectious disease in Jihe Hospital from Wuhan during January 24 to February 17. The information about patients’ age, gender, symptoms, temperature, use of TCMs and results of laboratory examinations during hospitalization were collected through hospital information system (HIS).

### Statistical analysis

The clinical characteristics of patients and frequency of CMs use were described. Independent t test was used to measure the differences of clinical characteristics among patients in varied demographic groups, and paired t test was used to measure the differences of patients’ blood test results between admission and discharge of hospital, which could indicate the effect of CMs. Correlation analysis was applied to investigate the relevance among various symptoms and TCM clinical features. Multiple linear regression analysis was applied to explore the factors affecting the length of hospital stay.

### Network pharmacology analysis

The compound information of all the 21 Chinese medicines in QFPDD were collected. There were one to three herbs selected as the sovereign medicinal (君药) to represent the main effect of the recommended formulas of five disease stages: mild, moderate, severe, critical and recovery stages.

For target prediction and active compound (C)—target (T) network construction, we input the SMILES of the compounds into online tools Similarity Ensemble Approach (SEA) to predict the putative targets. The software Cytoscape (version: 3.7.0) was used to construct active compounds-targets network, and the network parameters of each element were analysed based on the plug-in Network Analyzer for further analysis.

A web-based gene set analysis toolkit was applied for the Kyoto Encyclopedia of Genes and Genomes (KEGG) pathway enrichment analysis of putative targets. Parameter settings were as follows: Homo sapiens in Organism of Interest, Over-Representation Analysis (ORA) in Method of Interest and KEGG in Functional Database.

## Results

### Theoretic base of TCM comprehension on NCP

**Fig. 1:**
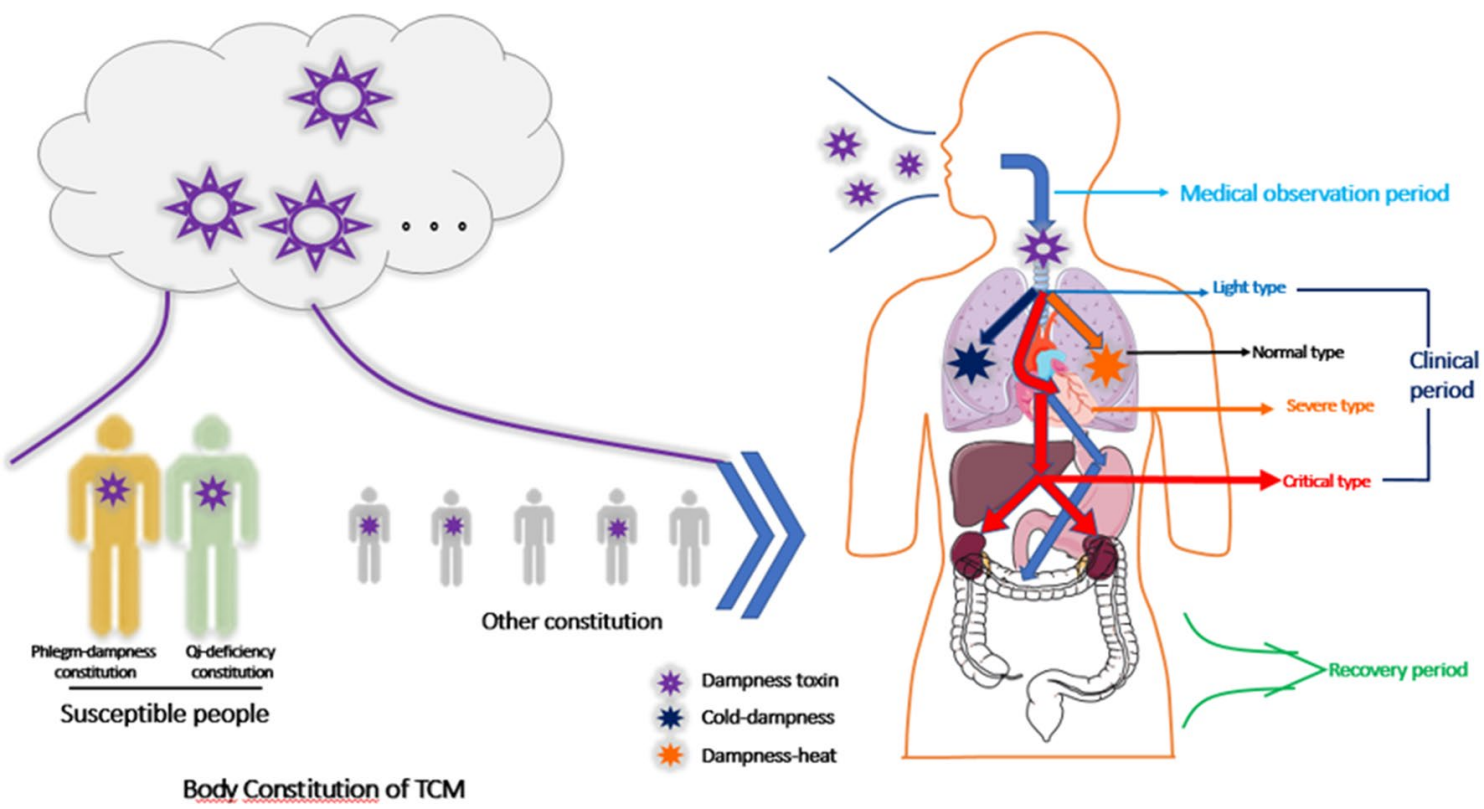
Diagram of pathological evolution of TCM

TCM comprehension on pathological evolution of NCP and medication paradigm were analyzed based on syndrome differentiation. As NCP belongs to the category of epidemic disease, the pathogen is generally attributed to dampness toxin according to the main symptom characteristics of this disease. TCM believes that NCP locates in lung, and is closely related to spleen and stomach, and its pathological changes involve in heart, liver and kidney in the later stages. Dampness pathogen can change into cold-dampness (寒湿) pathogen following the Yin body constitution (阴性体质), and also become the dampness-heat (湿热) pathogen following the Yang body constitution (阳性体质). Clinical observation shows that dampness toxin can directly invade into middle energizer (中焦) in partly NCP patients, and leads to the dysfunction of Qi movement. If the treatment method is appropriate and sufficient healthy Qi (正气) gradually recovers, the pathogen will be driven out, consequently the patient will enter into the recovery period. At the same time clinical manifestations appear some symptoms of Qi and Yin deficiency (气阴两虚证). Thus, the pathological evolution of NCP in TCM can be summarized as dampness toxin invading defense exterior (卫表) in early stage, and then enters the lungs and influences spleen function, eventually involves heart, liver and kidney, which causes Yang Qi collapse (阳气外脱) by excess pathogen and Yin and Yang separates from each other (阴阳离决). If the treatment is timely and suitable, sufficient healthy Qi can eliminate the pathogen, syndromes with deficiency of Qi and Yin in lung and spleen will be manifested (Fig. 1).

Although the pathogen between NCP and SARS have certain similarities in virus origination, the TCM pathogenesis of NCP and SARS appear to be different (Table 1).

**Table 1:** Characteristics of TCM Pathogenesis in NCP and SARS

	NCP (COVID-19)	SARS
Original area	Wuhan, Hubei	Foshan, Guangdong
Onset time	Nov, 2019	Nov, 2002
TCM pathogen	Dampness toxin	Epidemic toxin (疫毒)
Main symptoms	Low fever or no fever, some patients only felt fatigue, or dry cough, nausea, diarrhea. Severe dyspnea occurs 1 week later, which can lead to multiple organs failure.	The typical symptom is fever (frequent high fever), following fatigue, head and muscle pain. Some patients present dry cough, less sputum after 3 to 6 days, as well as chest discomfort and wheezing. In severe stage, the occurrence of gasp and acute respiratory distress.
Location of disease	Lungs, involving spleen and stomach, eventually affecting heart, liver and kidney	Mainly in lung
Pathological evolutional periods	1 Medical observation period: fatigue and gastrointestinal dysfunction or fever 2 Clinical period when diagnosed 2.1 Mild type: syndrome of cold dampness obstructing lung (寒湿郁肺), syndrome of lung with dampness heat retention (湿热蕴肺证) 2.2 Moderate type: syndrome of lung with dampness toxin retention (湿毒郁肺证), syndrome of cold dampness blocking lung (寒湿阻肺证) 2.3 Severe type: syndrome of pestilent toxin blocking lung (疫毒闭肺证), syndrome of dual blaze of Qi and blood (气血两燔证) 2.4 Critical type: syndrome of internal block and external collapse (内闭外脱证) 3 Recovery period: syndrome of Qi and Yin deficiency in lung and spleen (肺脾气阴两虚证)	1 Early stage: syndrome of epidemic toxin invading lung (疫毒犯肺证) 2 Progressive stage: syndrome of epidemic toxin obstructing lung (疫毒壅肺), syndrome of blocking lung with severe dyspnea (肺闭喘憋), syndrome of internal block and external collapse (内闭外脱证) 3 Recovery period: syndrome of Qi and Yin deficiency (气阴两虚证), syndrome of phlegm and blood stasis blocking collaterals (痰瘀阻络证).

Except using CMs treatment according to TCM different syndromes, the application of achievements from modern pharmacological research on CMs, targeting the pathological changes of NCP at different periods will also improve therapeutic effects. For example, *Ephedra sinica* Stapf, *Schizonepeta tenuifolia* Briq., Perillae Folium and Lonicerae Japonicae Flos have antipyretic and analgesic actions, and Ma Xing Shi Gan Decoction (麻杏石甘汤), *Arctium lappa* L. and *Poria cocos* (Schw.) Wolf. are able to regulate immune function and suppress inflammatory cytokine storm, suggesting that TCM plays a comprehensive beneficial regulating role in the treatment of NCP through multi-level and multi-pathways.

### Characteristics of patients and TCM utilization

Demographic characteristics of patients were shown in Table 2. The average age of all the 54 patients was 55.07 years old. Male patients (52.03 years old) were younger than female patients (60.25 years old) in average (*t *= 2.128, *P* = 0.028). The average length of hospital stay was 8.96 days. Patients over 45 years old stayed 9.79 days in hospital in average, which was longer than 7.64 days of patients under 45 years old (*t* = 2.232, *P* = 0.034).

**Table 2:** Demographic characteristics and TCM features of patients

Characteristic	Population
Gender	
Male	34
Female	20
Age	
< 45	11
[45, 60)	21
[60, 75)	18
≥ 75	4
Symptoms	
Fever	16
Cough	24
Shortness of breath	18
Abnormal digestion	5
Anorexia	8
Tongue manifestation	
Red tongue with white coating (舌红苔白)	24
Red tongue with yellow coating (舌红苔黄)	11
Others	16
Pulse manifestation	
Deep pulse (沉脉)	19
Slippery pulse (滑脉)	12
Others	20

The common symptoms were correlative with TCM clinical features (Table 3). Fever was negatively related to cough and shortness of breath, and was in positive correlation to abnormal digestion, Red tongue with white coating (舌红苔白) and deep pulse (沉脉). Cough was in negative correlation to shortness of breath and abnormal digestion. Shortness of breath was negatively related to deep pulse.

**Table 3:** Relevant of Symptoms and TCM clinical features

	Fever	Cough	Shortness of breath	Abnormal digestion	Anorexia	Red tongue with white coating	Deep pulse
Fever	1	− 0.299^a^	− 0.411^b^	0.346^a^	0.057	0.316^a^	0.450^b^
Cough	− 0.299^a^	1	− 0.203	− 0.311^a^	− 0.083	− 0.056	− 0.024
Shortness of breath	− 0.411^b^	− 0.203	1	0.032	0.02	− 0.15	− 0.406^b^
Abnormal digestion	0.346^a^	− 0.311^a^	0.032	1	0.039	− 0.016	0.234
Anorexia	0.057	− 0.083	0.02	0.039	1	− 0.17	− 0.175
Red tongue with white coating	0.316^a^	− 0.056	− 0.15	− 0.016	− 0.17	1	0.639^b^
Deep pulse	0.450^b^	− 0.024	− 0.406^b^	0.234	− 0.175	0.639^b^	1

^a^Correlation significant, at 0.05 level; ^b^at 0.01 level

NCP patients were prescribed with 87 kinds of CMs in total, and the most frequently used CMs were listed according to their classification (Table 4).

**Table 4:** The most frequently used CMs for NCP patients

Classification of TCM	Top 3 used TCMs	Frequency
Category of clearing heat and drying dampness and removing toxin (清热燥湿解毒类)	*Scutellaria baicalensis* Georgi (黄芩)	29
	*Lonicera japonica* Thunb (金银花)	11
	*Forsythia suspense* Vahl (连翘)	10
Category of aromatic herbs resolving dampness (芳香化湿类)	*Amomum villosum* Lour (砂仁)	28
	*Amomum compactum* (豆蔻)	12
	*Pogostemon cablin* (藿香)	9
Category of eliminating dampness with bland medicinal (淡渗利湿类)	*Poria cocos* (茯苓)	40
	*Alisma orientalis* (泽泻)	34
	*Coix lacryma*-*jobi* (薏苡仁)	36
Category of invigorating spleen and removing dampness (ISRD, 健脾祛湿类)	*Atractylodes macrocephala* (白术)	30
	*Astragalus membranaceus* (黄芪)	5
	*Dolichos* (白扁豆)	2
Category of power appetite and digestant medicinal (开胃消食类)	*Massa Medicata Fermentata* (神曲)	19
	*Hordeum vulgare* (麦芽)	18
	*Gallus gallus domesticus* (鸡内金)	13
Others	*Prunus armeniaca* (杏仁)	33
	*Paeonia suffruticosa* (丹皮)	27
	*Platycodon grandiflorum* (桔梗)	27

### Clinical effect of TCMs treatment

Multiple linear regression was employed with the length of hospital stay as dependent variable, and patients’ age, gender, symptoms and CMs they administrated as independent variables (Table 5). The method of backward elimination was chosen to get the optimal fitting degree of regression model (Table 6).

**Table 5:** Assignment of independent variables in multiple linear regression

Variables	Assignment
X1: Age	Continuous variable
X2: Gender	Male = 1, Female = 0
X3: Cough	Yes = 1, No = 0
X4: Short of breath	Yes = 1, No = 0
X5: Administrate category of clearing heat and drying dampness and removing toxin	Yes = 1, No = 0
X6: Administrate category of aromatic herbs resolving dampness	Yes = 1, No = 0
X7: Administrate category of ISRD	Yes = 1, No = 0
X8: Administrate other CMs	Yes = 1, No = 0

**Table 6:** Regression coefficients in multiple linear regression

Variable	Unstandardized coefficient		Standardized coefficient	*T*	*P*	Collinearity	
	B	S.E.	Beta			Tolerance	VIF
(Constant)	− 4.999	3.956		− 1.264	0.224		
X1:	0.106	0.054	0.446	1.963	0.067	0.522	1.914
X2:	4.538	1.565	0.727	2.899	0.010^*^	0.428	2.336
X3:	− 3.474	1.430	− 0.580	− 2.430	0.027^*^	0.474	2.110
X4:	− 2.665	1.311	− 0.436	− 2.033	0.059	0.586	1.707
X5:	1.925	1.442	0.309	1.335	0.201	0.504	1.983
X6:	− 1.510	1.409	− 0.242	− 1.072	0.300	0.529	1.892
X7:	7.864	2.291	1.051	3.433	0.003^**^	0.288	3.474
X8:	9.771	2.781	0.885	3.514	0.003^**^	0.425	2.355

*P < 0.5, **P < 0.01

The regression equation of length of hospital stay was:

$${\text{Y}}\,{ = }\, - \,4.999\, + \,0.106\;{\text{X}}_{1} \, + \,4.538\;{\text{X}}_{2} \, - \,3.474\,{\text{X}}_{3} \, - \,2.665\,{\text{X}}_{4} \, + \,1.925\,{\text{X}}_{5} \, - \,1.510\,{\text{X}}_{6} \, + \,7.864\,{\text{X}}_{7} \, + \,9.771\,{\text{X}}_{8} ,\,{\text{with}}\;{\text{R}}^{2} \, = \,0.569\,{\text{and}}\,{\text{adjusted}}\;{\text{R}}^{2} \, = \,0.353.$$

According to the standard coefficients, the factor affecting the length of hospital stay for the most was administrating category of ISRD, followed by administrating other CMs, male, and cough.

Patients received blood test both when admitted to hospital and discharge from hospital (Table 7).

**Table 7:** Values of blood test when admission and discharge of hospital

	Reference values	Observed values in average (95%CI)		*T*	*P*
WBC (*10^9^/L)	4–10	Admission	4.73 (3.97–5.49)	− 3.394	0.004^**^
		Discharge	6.68 (5.88–7.47)		
Neu %	50–70	Admission	71.41 (65.65–77.16)	0.319	0.754
		Discharge	71.62 (67.37–75.87)		
Lym %	20–40	Admission	21.30 (16.18–26.42)	0.016	0.987
		Discharge	20.28 (16.55–24.01)		
Mon %	3–12	Admission	6.55 (5.02–8.09)	− 0.561	0.583
		Discharge	6.90 (5.34–8.46)		
EOS %	0.5–5	Admission	0.54 (0.21–0.87)	− 2.800	0.013^*^
		Discharge	0.99 (0.70–1.28)		
Bas %	0–1	Admission	0.20 (0.12–0.28)	− 0.968	0.347
		Discharge	0.21 (0.14–0.29)		
Neu# (*10^9^/L)	2–7	Admission	3.45 (2.69–4.21)	− 2.359	0.031^*^
		Discharge	4.84 (4.06–5.62)		
Lym# (*10^9^/L)	0.8–4	Admission	0.94 (0.74–1.14)	− 2.948	0.009^**^
		Discharge	1.30 (1.09–1.51)		
Mon# (*10^9^/L)	0.12–1.2	Admission	0.31 (0.22–0.40)	− 1.897	0.076
		Discharge	0.46 (0.32–0.61)		
Eos# (*10^9^/L)	0.02–0.5	Admission	0.02 (0.01–0.04)	− 4.592	0.000^***^
		Discharge	0.06 (0.05–0.08)		
Bas# (*10^9^/L)	0–0.1	Admission	0.00 (0.00–0.01)	− 3.846	0.001^**^
		Discharge	0.01 (0.01–0.02)		
RBC (*10^12^/L)	4–5.5	Admission	4.47 (4.21–4.73)	4.971	0.000^***^
		Discharge	4.03 (3.76–4.30)		
HGB (g/L)	120–160	Admission	137.40 (128.85–145.95)	5.067	0.000^***^
		Discharge	123.53 (114.27–132.80)		
HCT (%)	35–51	Admission	42.12 (39.52–44.72)	4.972	0.000^***^
		Discharge	38.05 (35.37–40.72)		
MCV (fL)	80–100	Admission	94.29 (92.54–96.04)	− 0.469	0.645
		Discharge	94.38 (92.86–95.90)		
MCH (g/L)	320–360	Admission	326.13 (322.83–329.44)	1.070	0.301
		Discharge	324.40 (319.66–329.14)		
PLT (*10^9^/L)	100–300	Admission	186.80 (150.28–223.32)	− 4.598	0.000^***^
		Discharge	299.80 (261.20–338.40)		
MPV (fL)	6.5–12	Admission	9.00 (8.55–9.45)	5.009	0.000^***^
		Discharge	8.27 (7.82–8.71)		
PDW (%)	9–17	Admission	16.00 (15.79–16.21)	1.464	0.164
		Discharge	15.87 (15.67–16.06)		
PCT (%)	0.108–0.280	Admission	0.16 (0.14–0.19)	− 4.014	0.001^**^
		Discharge	0.25 (0.22–0.28)		

*P < 0.5, **P < 0.01, ***P < 0.001

The indicators of blood test included white cell count (WBC), percentage of neutrophils (Neu %), percentage of lymphocytes (Lym %), percentage of mononucleosis (Mon %), percentage of eosinophils % (EOS %), percentage of alkaline granulocytes (Bas %), number of neutrophils (Neu#), number of lymphocytes (Lym#), number of single-core cells (Mon#), number of eosinophils (Eos#), number of alkaline granulocytes (Bas#), red cell count (RBC), hemoglobin concentration (HGB), hematocrit (HCT), average red blood cell volume (MCV), average red blood cell hemoglobin concentration (MCH), platelet number (PLT), average platelet volume (MPV), platelet distribution width (PDW) and platelet pressure (PCT).

(Table 7 Values of blood test when admission and discharge of hospital)

By comparing the parameters of blood test between admission and discharge of hospital, the values of WBC, EOS %, Neu#, Lym#, Eos#, Bas#, PLT and PCT increased, while values of RBC, HGB, HCT, and MPV decreased. The result indicated that TCM treatment significantly ameliorated the immune ability against SARS-CoV-2 in patients.

### Network pharmacology of QEPDD and NHC-recommended formulas

The five disease stages consist of nine syndromes and nine formulas. The sovereign medicinal of mild disease stage contained *Pogostemon Cablin (Blanco), Atractylodes Lancea (Thunb.)Dc., Scutellariae Radix, Chaihu Radix Bupleuri, Forsythiae Fructus*; the moderate stage contained gypsum, *Atractylodes Lancea (Thunb.)Dc., Polygoni Cuspidati Rhizoma Et Radix, Pogostemon Cablin (Blanco), Verbenae Herb*; the severe stage contained *Ephedra Herba,* gypsum*, Lepidii Semen Descurainiae Semen*, buffalo horn; the critical stage contained *Panax Ginseng C. A. Mey., Aconiti Lateralis Radix Praeparata*; and the recovery stage contained *Hedysarum Multijugum Maxim., Ophiopogon japonicus (Linn. f.) Ker*-*Gawl, Panacis Quinquefolii Radix*.

There are 21 CMs in the QFPDD, and 16 CMs in recommended formulas of five disease stages. After deleting duplication, 32 different CMs were selected in total. As the *Ophiopogon japonicus (Linn. f.) Ker*-*Gawl.*, gypsum, and buffalo horn were not found in TCMSP database, finally 29 CMs were picked in this study. There are 201 compounds in recommended formulas of five disease stages and 288 compounds in the QFPDD after screening. Comparing with the compound in sovereign medicinal, we discovered kaempferol, beta-sitosterol, Stigmasterol, quercetin, luteolin, Genkwanin, diop, isorhamnetin participated in three or more disease stages. At the same time, these compounds were not unique to a single Chinese herbal medicine, but many common CMs. Comparing the compounds in QFPDD with those in recommended formulas of the first four disease stages, seven of the eight compounds (except isorhamnetin) were found to be representative in QFPDD and in recommended formulas of three or four disease stages.

The recommended formulas included 164 types of putative targets in mild disease stage, 147 types in moderate stage, 150 types in severe stage, 88 types in critical stage and 112 types in recovery stage. Totally, there were 204 types of different targets in recommended formulas, and 240 targets in QFPDD. After comparing the putative targets of five disease stage, it was found that 169 of the 204 targets were common targets, among which 58 were involved in the treatment of all five disease stages. By comparing QFPDD with recommended formulas of the first four stages, it was found that only 9 of 248 targets have nothing to do with QFPDD, and 60 targets were common targets.

**Fig. 2:**
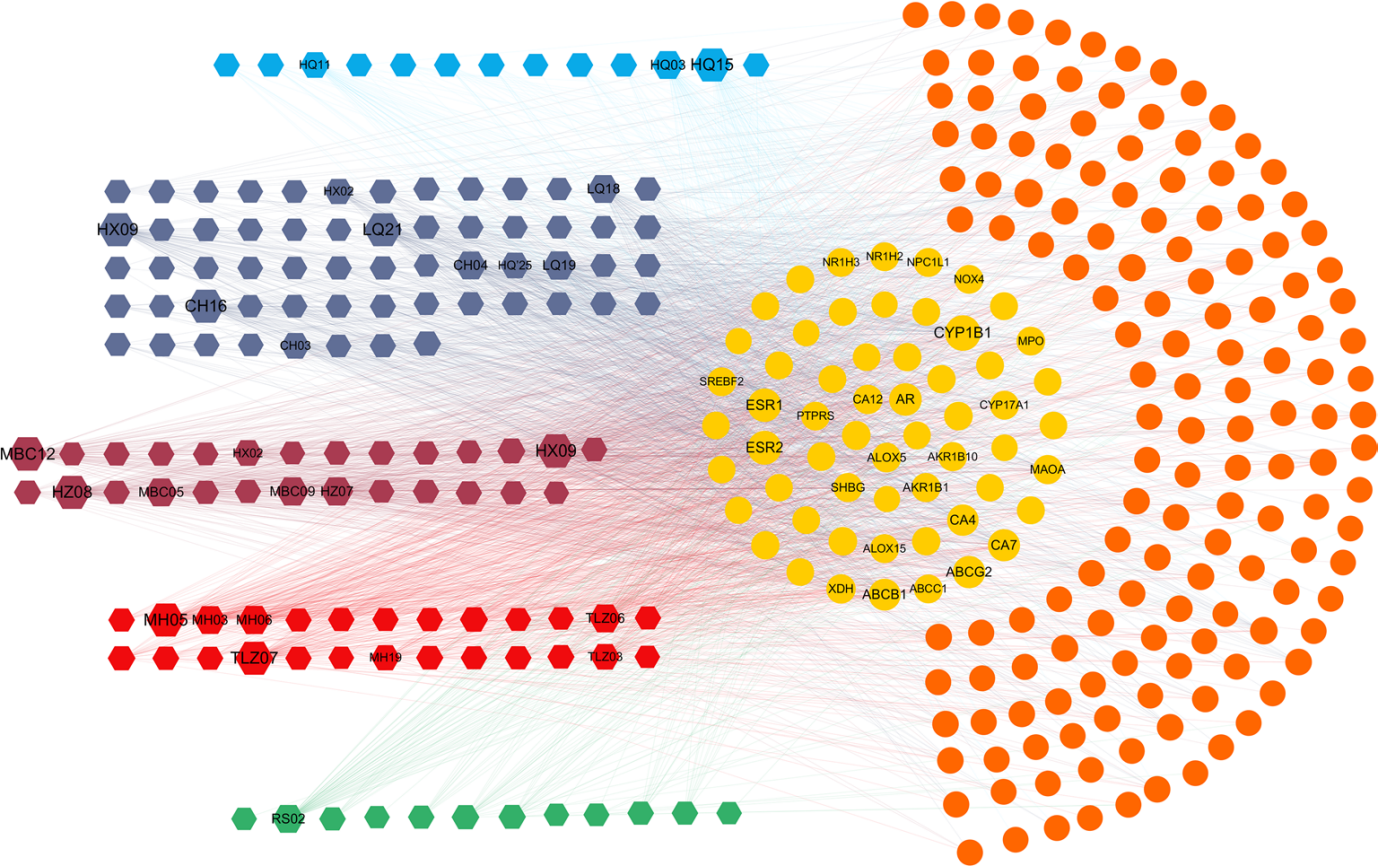
Active compounds (C)-putative targets (T) network of five disease stages

According to the compounds (C)-putative targets (T) network (C-T network) related to the five stages, the degree value of quercetin, luteolin, kaempferol, acacetin and genkwanin were all greater than 30 and exist in a variety of CMs, which acted on different disease stages (Fig. 2). And the degree value of a total of 25 targets was greater than 30, among which the first five were CYP1B1, ESR1, ESR2, AR, and ABCG2, respectively.

**Fig. 3:**
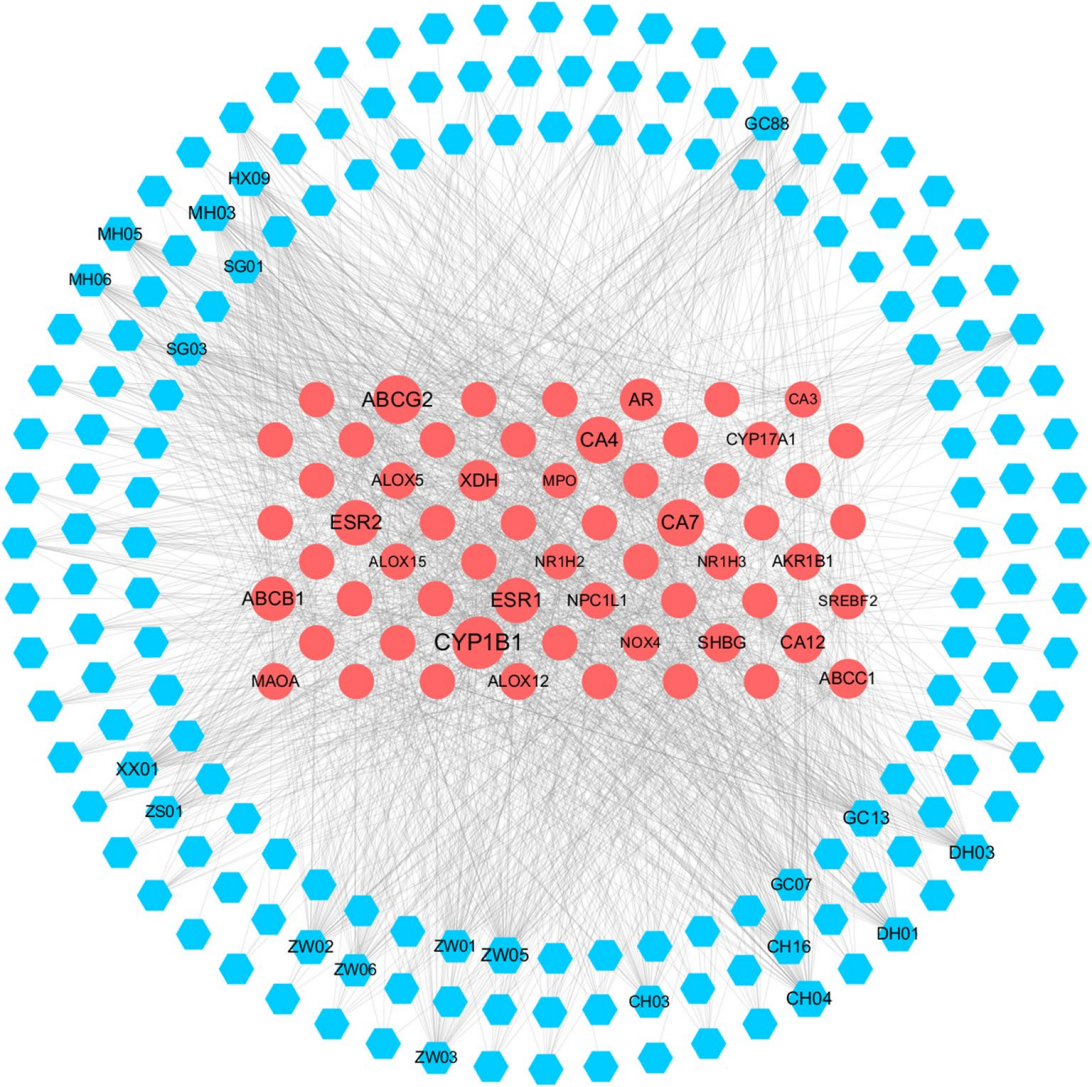
Active compounds (C)-putative targets (T) network of QFPDD

Then a C-T network of QRPDD was constructed similarly. By considering the large number of targets, only 60 common targets in QFPDD and recommended formulas of the first four disease stages were selected for visualization (Fig. 3). For the compounds, kaempferol, quercetin, luteolin, galangin, luteolin, isorhamnetin and the degree value were all greater than 30. For the targets, the first five were CYP1B1, ABCG2, CA7, CA4 and ESR2, respectively.

The hexagon on the left represents the compounds, and the different colours from top to bottom represent the different compounds in mild, moderate, severe, critical and recovery stages of the disease. The right circle represents the putative targets, the yellow represents the 58 common targets of the five disease stages, and the orange represents 197 non-common targets. The edges show the relationship between the compounds and putative targets obtained by SEA prediction. Size of the point is related to its degree value, and the larger the value, the larger the point.

The blue hexagon represents the compound, and the red circle points represent the 60 targets shared by QFPDD and the recommended compound of the first four disease stages. The rest is consistent with Fig. 2.

**Fig. 4:**
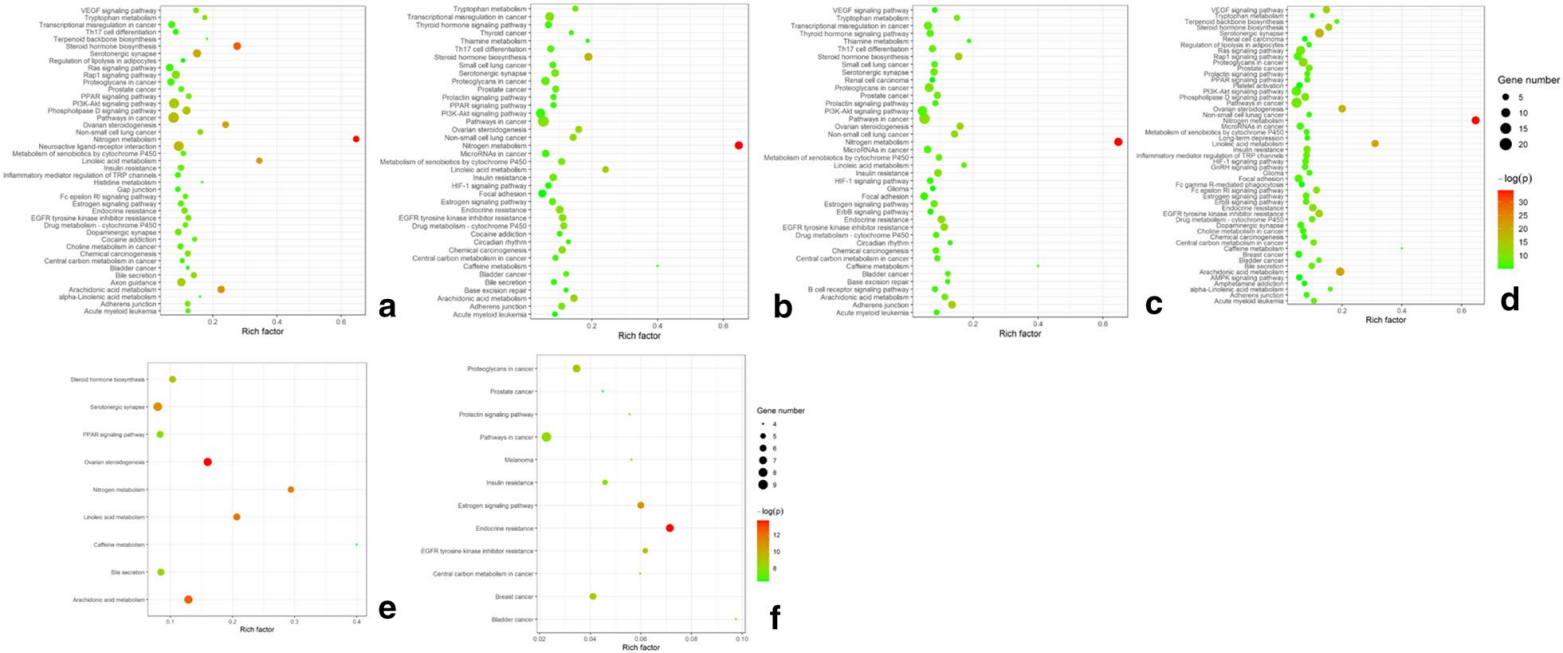
The bubble diagram of KEGG pathway enrichment analysis

As shown in Fig. 4, according to the enrichment results of pathways corresponding to the five disease stages, figure B, C and D show a large number of enrichment pathways, while E and F are more concentrated. Nitrogen metabolism is presented in the first four diseases stages, with a high enrichment rate as well as a low *p* value, and the expression of arachidonic acid metabolism in the critical disease stages was the most obvious, indicating that formulas were more inclined to inhibit pathogens in the critical diseases stage. Ovarian steroidogenesis exists mainly in severe and critical disease stages. For QFPDD, nitrogen metabolism, linoic acid metabolism and steroid hormone biosynthesis have high enrichment rate (Figure A), which is roughly consistent with the results of mild and moderate stages.

Figure A represents QFPDD and figure B-F represent recommended formulas of mild, moderate, severe, critical and recovery stages of the disease, respectively. The x axis means enrichment ratio and the y axis means the name of KEGG pathways.

## Discussion

### Efficacy of TCM treatment on NCP

Generally, the clinical results of our study showed that 54 NCP patients enhanced immune ability against COVID-19 by detecting blood samples after TCM treatment and category of ISRD appears to shorten patients’ hospitalization days.

Firstly, the hemogram changes of patients in this study can reflect the efficacy of TCM treatment on NCP in a certain extent. As a common symptom among NCP patients, the lymphocytopenia was observed in this study, though it was not severe. According to recent studies on clinical characteristics of NCP, lymphocytopenia was one of the most common laboratory abnormalities observed in NCP patients, and occurred in 70.3–83.2% of patients [8, 9]. The abnormalities like lymphocytopenia suggested that COVID-19 infection may be associated with cellular immune deficiency [9], thus the increase of lymphocyte during the TCM treatment in this study could be regarded as a result of TCM improving patients’ hemogram by adjusting immune system [10].

In this study, the average hospitalization time of 54 NCP patients in moderate type was 8.96 days under the treatment of TCM, and the length of hospital stay was related to the TCM in category of ISRD, suggesting that Chinese medicines could effectively shorten the pathological evolution of NCP. As the pathogenesis of NCP in Chinese medicine theory belongs to dampness and toxin, and dampness pathogen mainly influence the digestive function of spleen, stomach and intestine, the primary therapeutic strategy focuses on improving digestive function for dispelling dampness pathogen.

Additionally, our study also found that abnormal digestion was associated with the clinical symptoms of fever and cough by correlation analysis, which powerfully illustrated the relationship of the pathogenesis of NCP with the dysfunction of digestive system (e.g. spleen, stomach and intestine). Therefore, our study demonstrates that Chinese medicines with an effect of ISRD should be beneficial to attenuate the pathological evolution of NCP.

Interestingly, the category of removing toxin did not account for the predominant relation with the length of hospital stay from the result of multiple linear regression analysis, showing the therapeutic methods were primarily attributed to ISRD rather than removing toxin. The results indicated the function of Chinese medicines on NCP treatment focus on strengthening sufficient healthy Qi (enhancing body immune ability) instead of dispelling pathogen (targeting against COVID-19). One of supported evidences was that the number of leukocytes, neutrophil, lymphocyte and platelet significantly increased at the end of integrated treatment.

### Pathological mechanism of anti-NCP TCM

During rescuing critical NCP patients in Wuhan, Academician Li Lanjuan’s team mentioned that it was important to maintain patients’ intestinal microenvironment balance, and TCM had advantages on reducing intestinal bacterial migration to lung and secondary infection. This view has been included into the Diagnosis and Treatment Guideline of NCP (Edition 7) [7]. As the development of intestine and lung originate from the same source in embryonic stage, studies indicate that bacterial and viral infections in lung can affect intestinal microenvironment through the “Gut-lung axis”. And vice versa, in animal model of sepsis and acute respiratory distress syndrome, the intestinal specific bacteria (such as bacteroides spp) can directly transfer to lung through damaged intestinal barrier, revealing that these two critical and high mortality diseases have a common pathogenesis mechanism [11]. Sepsis and acute respiratory distress syndrome are also the pathological results of severe and critical NCP patients suffering from cytokine storm, which are the main death cause for patient [12, 13]. Previous studies have confirmed that intestinal microenvironmental disorders and imbalance of intestinal flora can cause the occurrence and development of respiratory diseases through the Gut-lung axis. Therefore, lung diseases can be treated by adjusting intestinal microbiota [14, 15].

In the theoretical system of TCM, there is a doctrine of “lung and large intestine in pair (肺合大肠)”. Some scholars have suggested that TCM pathogenesis of NCP is related to the “lung-spleen-large intestine” dysfunction [10, 16, 17]. It has been discovered that Houttuynia cordata polysaccharide (HCP), a Chinese herbal medicine extract from *Houttuynia cordata* Thunb. can keep the intestinal homeostasis in H1N1 virus-infected mice, and protect the intestinal wall barrier, inhibit IL-1β production mediated by Toll receptors, and improve the expression of IL-10, suggesting that the effect of anti-inflammatory damage is related to regulation of intestinal microbiota. As a main active ingredient of *Scutellaria baicalensis* Georgi and *Coptis chinensis* Franch., Berberine’s bioavailability and in vivo metabolism are found to have close relationship with intestinal organic acids and microbiota [18, 19]. The doctrine of lung and large intestine in pair in TCM theory is similar to the Gut-lung axis in the theory of modern medicine. The most frequently used CMs for NCP patients in our study also suggests channel tropism (归经) of numerous CMs belongs to lung, spleen, stomach and intestine. Therefore, CMs can achieve the purpose of relieving and curing lung diseases by adjusting the intestinal microenvironment balance. Moreover, it is not only one of the mechanisms of Chinese medicine treatment for NCP, but also one important approach to screen novel Chinese medicines for treating NCP in the future.

The network pharmacology results in this study indicated that the recommended formulas in five disease stages have both generality and individuality in the compound level, in which compound quercetin, luteolin, kaempferol, acacetin etc., all are involved in the treatment of multi-stages of the disease. The target and KEGG pathway enrichment results also show the certain integrity and the different tendency of formulas. Meanwhile, by comparing the QFPDD with the recommended formula of the first four stages [20, 21], we discover that the QFPDD has a certain integrity on the compound level. However, on the target level, the QFPDD has covered most of the predicted target of the recommended formula of the first four disease stages, and the mutual target from both two sources have a higher network parameter. The KEGG pathways enrichment results about QFPDD are consist with the effects of recommended formulas of mild and moderate stages as well. All these results confirmed the consistency of QFPDD with the recommended formulas of the first four disease stages from the perspective of network pharmacology.

In summary, this study suggests TCM has a systemic theoretical understanding on the pathological evolution of NCP (COVID-19), and the symptom positive correlation between fever and abnormal digestion indicates that researchers should pay more attention on intestinal functional recovery and microenvironmental balance in the treatment of NCP. Additionally, the network pharmacology results demonstrate the appropriateness of theatrical formulas for QFPDD and other recommended formulas in guideline, for the compounds corresponding to targets can cover various stages of the disease.

### Advantages and implications of TCM utilization

As there has been no effective targeted drugs for NCP up to now, the early intervention to NCP is important in controlling the spread of COVID-19. According to the Protocol for Prevention and Control of COVID-19 (Edition 6) by Chinese Center for Disease Control and Prevention, the use of TCM in the prevention and treatment of infectious diseases was encouraged and supported, and TCM was applied in the whole process of disease [7, 22].

Numerous data have showed that the early intervention of TCM and modern medicine have positive effects on shortening time of hospitalization and ameliorating symptoms, reducing the development of mild and moderate case to severe case and the mortality rate, improving the cure rate and the rehabilitation of the recovery population of NCP patients [23]. A report from Hunan province suggested that due to the increasing the use of TCM for NCP, the patients’ average hospitalization day was shortened for 2 days, and none of the patients turned to critical stage [15]. A study about 34 NCP patients (mostly moderate type) from Wuhan found satisfied efficacy of integrated use of CM and western medicine on protecting heart, liver, kidney and other organ functions, as well as reducing inflammatory response and improving immune function, through the observation of patients treated with conventional western medicine, CM decoctions, traditional Chinese patent medicines and CM injections [24, 25]. In a study on 6 cases of severe and critical NCP, the results showed that Qufeidu No.1 Formulae (祛肺毒一号方) combined with conventional western medicine treatment, as well as integrating Xuebijing injection (血必净注射液) and/or Lianhua Qingwen capsule (连花清瘟胶囊) significantly improved pathological evolution of critical patients, consequently all of patients recovered [26, 27].

However, there were still deficiencies of the clinical research evidence on the integration of traditional Chinese and western medicine treatment for NCP, mainly embodying small sample size, the absence of control group, and the diversification of treatment programs. Therefore, constantly expanding the sample size and employing standardized randomized controlled trial (RCT) design will give more convincing scientific evidence to demonstrate the superiority of TCM for NCP treatment.

**Fig. 5:**
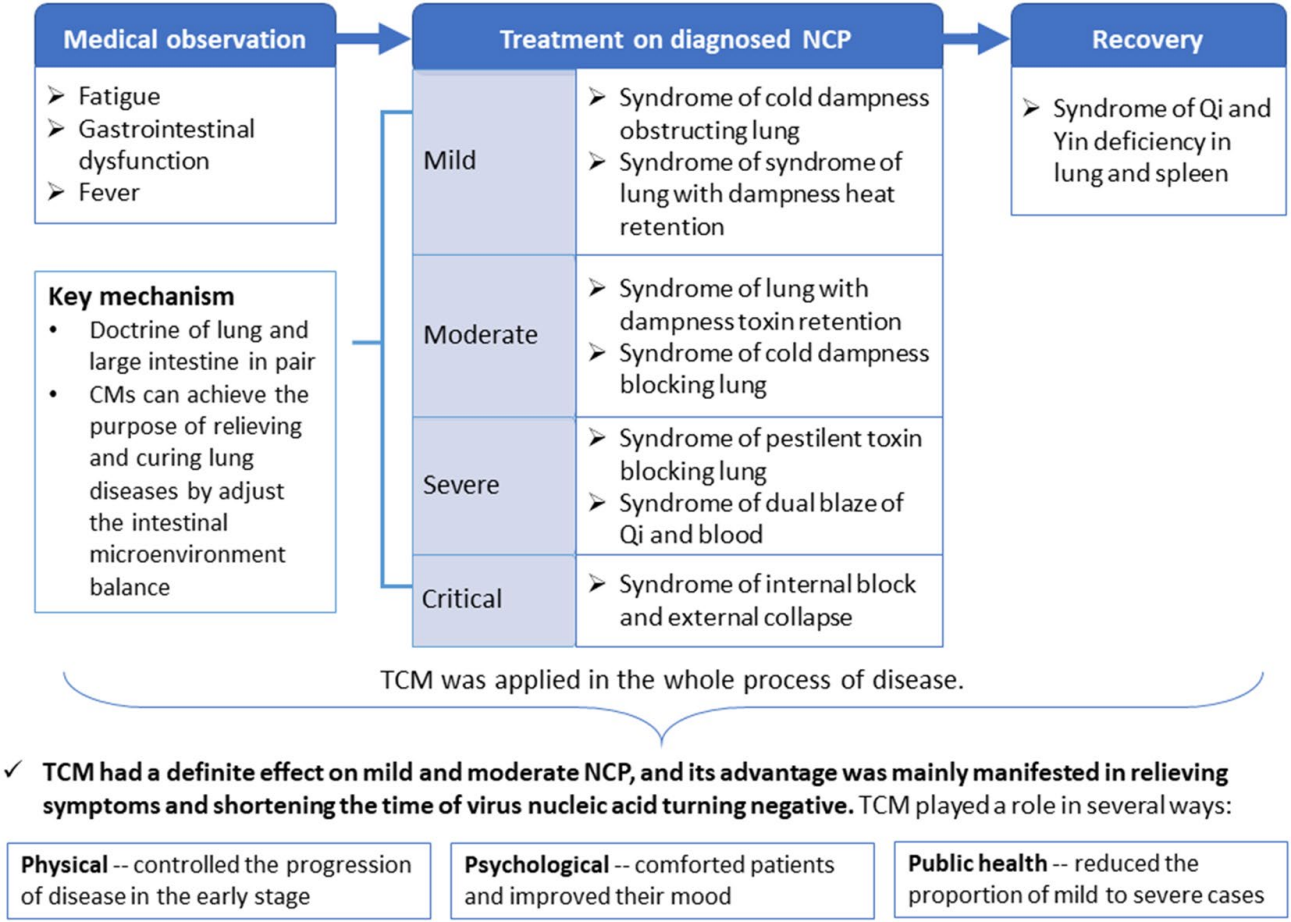
The major advantages and key mechanisms of TCM on the treatment of NCP

At present, NCP has been nearly controlled in China. By the end of April 1st, 2020, up to 92.44% of NCP patients have recovered in China. On March 23, the state council information office of China reported that 74,187 confirmed COVID-19 patients (91.5% of total confirmed cases) accepted the combined treatment with TCM and modern medicine, and the clinical efficacy observations showed that the total effective rate reached more than 90% [28].

As Tong Xiaolin said, practice proved the importance of sticking to both TCM and modern approaches, and Chinese medicine had the unique advantage in establishing a collaboration mechanism between them [29]. In summary, TCM had a definite effect on mild and moderate NCP, mainly manifested in relieving symptoms and shortening the time of virus nucleic acid turning negative (Fig. 5). Moreover, TCM not only played a role in controlling the progression of disease in the early stage, but also comforted patients psychologically [30]. In addition, TCM reduced the proportion of mild to severe cases, which could contribute to save medical resources from the perspective of public health.

The utilization of TCM has implications for the development of medicines in the future. As the NCP virus has some mutations when compared to SARS or MERS coronavirus, the natural compounds effectiveness against the two previous coronaviruses might not be present in the new virus, which urges updated TCM formulas. In this study, the anti-NCP effects of the natural compounds have been mainly confirmed by the screening method, and the treatment effect of dampness pathogen results in the shortening of hospital stay for patients of mild type. Thus, TCM treatment was not only effective for the NCP patients, but also advantageous to the clinical development of medicines to dampness pathogen and lung infection diseases, which have high incidence rate of respiratory system syndrome. Therefore, Chinese medicine treatment has positive impact on infectious lung diseases, as well as the development of new medicines.

## Conclusion

Traditional Chinese medicine has a systemic theoretical understanding on the pathological evolution of novel coronavirus pneumonia and plays a positive role in NCP treatment according to the clinical data from Wuhan in this study. Multiple linear regression analysis shows that the TCM category of invigorating spleen and removing dampness improved patients’ recovery, suggesting the vitalness of regulating intestinal function and keeping microenvironmental balance in the TCM treatment of novel coronavirus pneumonia. The network pharmacology results demonstrate active compounds from QFPDD and recommended formulas by National Health Commission contribute to recovery of different disease progresses during TCM treating novel coronavirus pneumonia. Chinese medicine treatment is advantageous in treating NCP, as well as the development of new medicines for infectious lung diseases.

## Data Availability

The datasets analyzed during the current study are available from the corresponding author on reasonable request.

## Abbreviations

Bas: Alkaline granulocytes
CM: Chinese medicine
EOS: Eosinophils
HCT: Hematocrit
HGB: Hemoglobin concentration
HIS: Hospital information system
KEGG: Kyoto encyclopedia of genes and genomes
Lym: Lymphocytes
MCH: Average red blood cell hemoglobin concentration
MCV: Average red blood cell volume
Mon: Mononucleosis
MPV: Average platelet volume
NCP: Novel coronavirus pneumonia
Neu: Neutrophils
NHC: National Health Commission
ORA: Over-representation analysis
PCT: Platelet pressure
PDW: Platelet distribution width
PLT: Platelet number
QFPDD: Qingfei Paidu decoction
RBC: Red cell count
SARS: Severe acute respiratory syndrome
SEA: Similarity ensemble approach
TCM: Traditional Chinese medicine
WBC: White cell count
